# Notch Signaling Coordinates Progenitor Cell-Mediated Biliary Regeneration Following Partial Hepatectomy

**DOI:** 10.1038/srep22754

**Published:** 2016-03-08

**Authors:** Jie Lu, Yingqun Zhou, Tianyuan Hu, Hui Zhang, Miao Shen, Ping Cheng, Weiqi Dai, Fan Wang, Kan Chen, Yan Zhang, Chengfeng Wang, Jingjing Li, Yuanyuan Zheng, Jing Yang, Rong Zhu, Jianrong Wang, Wenxia Lu, Huawei Zhang, Junshan Wang, Yujing Xia, Thiago M. De Assuncao, Nidhi Jalan-Sakrikar, Robert C. Huebert, Bin Zhou, Chuanyong Guo

**Affiliations:** 1 Department of Gastroenterology, Shanghai 10th People’s Hospital, Tongji University School of Medicine, Shanghai, People’s Republic of China; 2 Institute for Nutritional Sciences, Shanghai Institutes for Biological Sciences, Chinese Academy of Sciences, Shanghai, People’s Republic of China; 3 Division of Gastroenterology and Hepatology; Mayo Clinic and Foundation, Rochester, MN, USA.

## Abstract

Aberrant transcriptional regulation contributes to the pathogenesis of both congenital and adult forms of liver disease. Although the transcription factor RBPJ is essential for liver morphogenesis and biliary development, its specific function in the differentiation of hepatic progenitor cells (HPC) has not been investigated, and little is known about its role in adult liver regeneration. HPCs are bipotent liver stem cells that can self-replicate and differentiate into hepatocytes or cholangiocytes *in vitro*. HPCs are thought to play an important role in liver regeneration and repair responses. While the coordinated repopulation of both hepatocyte and cholangiocyte compartment is pivotal to the structure and function of the liver after regeneration, the mechanisms coordinating biliary regeneration remain vastly understudied. Here, we utilized complex genetic manipulations to drive liver-specific deletion of the Rbpj gene in conjunction with lineage tracing techniques to delineate the precise functions of RBPJ during biliary development and HPC-associated biliary regeneration after hepatectomy. Furthermore, we demonstrate that RBPJ promotes HPC differentiation toward cholangiocytes *in vitro* and blocks hepatocyte differentiation through mechanisms involving Hippo-Notch crosstalk. Overall, this study demonstrates that the Notch-RBPJ signaling axis critically regulates biliary regeneration by coordinating the fate decision of HPC and clarifies the molecular mechanisms involved.

Parenchymal cell loss, impaired liver function, and extracellular matrix deposition are common end-points in a variety of hepatocellular disorders as well as the cholangiopathies^1^. Furthermore, while the liver is known for its remarkable capacity to regenerate following partial hepatectomy (PHx) or other injuries^2^, the intrinsic regenerative capacity of the liver is impaired in the setting of these chronic diseases^3^. Therefore, studying the pathogenic mechanisms of acute and chronic liver diseases and the regeneration mechanisms after injury will be critical to emerging treatment strategies.

During normal regeneration, the various cell types in the liver undergo controlled hypertrophy and proliferation, resulting in the growth of the remnant liver lobes to precisely restore the pre-PHx liver mass. The roles of various growth factors, cytokines, and receptors in liver regeneration have been extensively studied for many decades^4^. However, despite the impressive regenerative potential of the liver resulting from the mitogenic capacity of hepatocytes and cholangiocytes, when this parenchymal replication is overwhelmed in the context of chronic liver disease, an aberrant form of liver regeneration occurs which fails to reconstitute liver synthetic function and tends to promote fibrosis and chronic disease progression^5^.

The intrahepatic biliary system appears to play an important role in liver regeneration, especially since the terminal bile ductules (the canals of Hering) are a site that comprises a niche for hepatic progenitor cells (HPCs). This bipotent cell population (also referred to as oval cells in rodents) is barely identifiable in normal livers, but dramatically expands following certain forms of liver injury^6^. Historically, it has been assumed that, depending upon the type and severity of liver damage, including whether it is acute or chronic, HPCs may differentiate into mature hepatocytes or cholangiocytes. This is based on the stem-cell characteristics of these cells when isolated, including an ability to self-replicate and to maintain a bipotential differentiation capacity *in vitro*. In human disease, HPCs form a central component of the ductular reaction in which HPCs expand to form an activated niche. Whether this process contributes directly to the reconstruction and repair of damaged hepatocytes *in vivo* remains a topic of debate since some lineage tracing studies suggest that this is the case^7^ while others suggest that regenerated hepatocytes derive exclusively from parenchymal hepatocytes^8^,^9^,^10^,^11^ or even from mature biliary cells^12^,^13^. However, the contribution of HPCs to biliary regeneration has not yet been clearly delineated.

The Notch signaling pathway is a highly conserved signal transduction mechanism that is essential for normal embryonic development, cellular proliferation, specification, and differentiation in organisms as diverse as nematodes, insects, and mammals^14^. In mammals, the canonical Notch pathway includes four receptors, Notch 1, 2, 3 and 4 (N1, N2, N3, and N4) and two families of ligands: Jagged-1 and 2 (JAG1, JAG2); and Delta-like-ligand 1, 3, and 4 (Dll1, Dll3, and Dll4). The Notch signaling pathway involves the ligand-induced activation of the Notch receptor, its proteolytic cleavage, and the subsequent translocation of its intracellular domain to the nucleus, where it acts as a transcriptional regulator. Upon ligand binding, the Notch receptor undergoes sequential proteolysis, releasing the intracellular domain, which translocates to the nucleus and associates with recombination signal-binding protein immunoglobulin kappa J (RBPJ). Interaction with the Notch intracellular domain typically converts the RBPJ corepressor complex into a coactivator complex and mediates gene transcription^15^. Human genetic diseases and mutant mouse models have clearly illustrated the importance of Notch signaling in the specification and remodeling of the intrahepatic bile ducts (IHBD). Indeed, Alagille syndrome is an autosomal dominant disorder in humans caused by mutations in the Notch ligand JAG1 or less commonly, in the Notch receptor N2^16^,^17^,^18^.

RBPJ is a common downstream transcription factor for each Notch receptor, and its absence results in completely abrogated Notch signaling. Several recent studies have assigned Notch important roles in liver regeneration, the regulation of ductal plate formation, and biliary morphogenesis in mice^19^. Global *Rbpj* gene KO can lead to liver sinusoidal endothelial cell proliferation, vascular occlusion, whole-liver congestion, and sinus cavity edema^20^, complicating analysis of its effects on HPCs. Therefore, cell type-specific KO studies are needed, such as those pursued here.

In this study, we hypothesized that Notch–RBPJ regulates bile duct regeneration by coordinating the proliferation and differentiation of HPCs toward cholangiocytes. To test this hypothesis, we used multiple *in vitro* and *in vivo* models including HPC lines, isolated HPCs, and complex murine genetic models (for cell type-specific gene deletion and lineage tracing). *In vivo*, we specifically analyzed the phenotype of liver-specific RBPJ knockout by evaluating biliary development and biliary regeneration after PHx. We report that the conditional deletion of RBPJ, the common downstream transcription factor for Notch receptors, impairs the processes of biliary development as well as biliary regeneration after PHx. Our *in vitro* studies define the role of Notch-RBPJ in regulating biliary differentiation from HPCs and identify an RBPJ/NICD co-repressor complex that inhibits YAP transcription and hepatocyte differentiation. This suggests Hippo-Notch crosstalk as a critical regulatory node in HPC differentiation. This study not only extends our understanding of the molecular regulatory machinery involved in liver regeneration, but also provides novel therapeutic targets in liver disease for further study.

## Materials and Methods

### Mice

The RBPJ^floxed^Rosa26^mTmG/+^Rosa26^laz/+^ Alb–Cre mouse strain was a gift from the Binzhou Laboratory (Institute for Nutritional Sciences, Shanghai) and was maintained in our laboratory. The Mx1–Cre transgenic mouse, in which the *Cre* gene is under the control of the poly(I:C)-inducible *Mx1* promoter, was purchased from the Model Animal Research Center of Nanjing University. All these mouse strains were raised in our laboratory and all animal experiments were performed according to protocols approved by the Institutional Animal Care and Use Committee of Tongji University.

**Figure 1:**
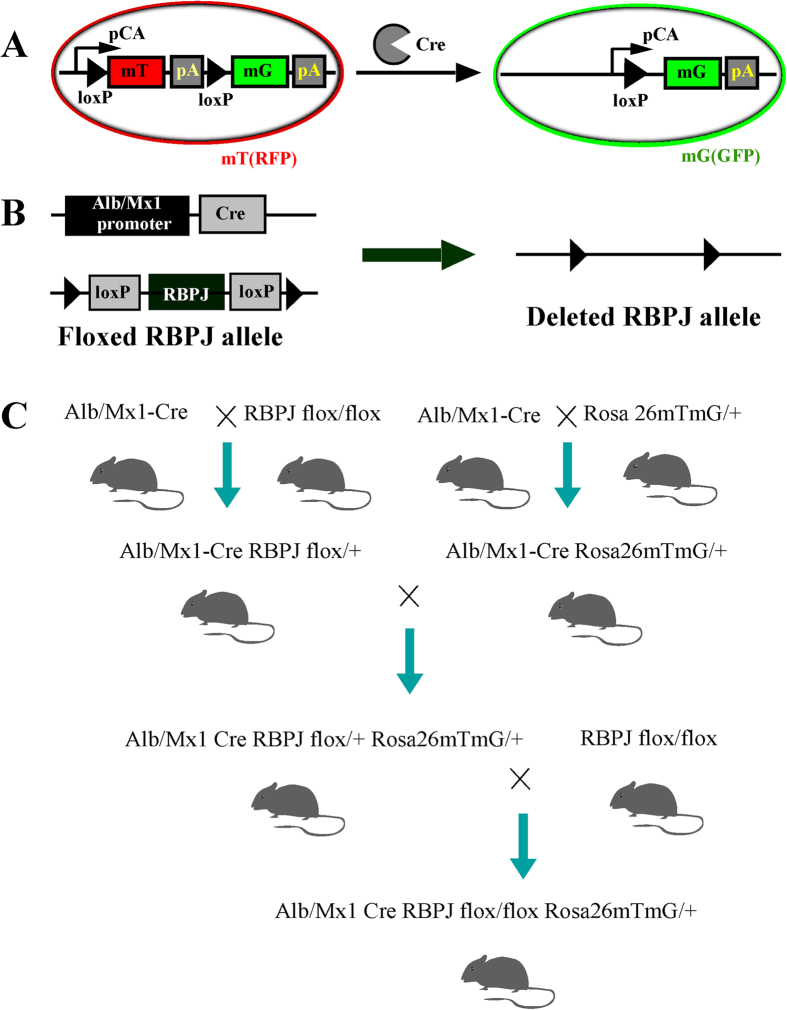
Transgenic mouse design strategy. (**A**) Cre–Loxp system, the mT gene expresses red fluorescent protein in the absence of Cre and the mG gene expresses green fluorescent protein in the presence of Cre. (**B**) Using the Cre–Loxp system, we constructed liver-specific *Rbpj*-KO mice by crossing the liver specific Cre with RBPJ flox/flox mice. (**C**) Using a sequential breeding strategy, we constructed liver-specific *Rbpj-*KO mice expressing the Rosa26^mtmG/+^ genetic marker.

The Alb–Cre and Mx1–Cre mice were crossed with Rosa26^laz/+^Rosa26^mTm G/+^ mice to produce Alb–Cre–Rosa26^mTm G/+^, Mx1–Cre–Rosa26^mTm G/+^, Alb–Rosa26^mTm G/+^ and Mx1–Cre–Rosa26^mTm G/+^ mice. These mice were then crossed with RBPJ^flox/flox^ mice to produce heterozygous and homozygous mice. After multiple rounds of crossing, we obtained the following genotypes which were used in this study: RBPJ^flox/+^–Alb–Cre–Rosa26^mTmG/+^, RBPJ^flox/flox^–Alb–Cre–Rosa26^mTmG/+^, RBPJ^flox/+^–Mx1–Cre–Rosa26^mTmG/+^, RBPJ^flox/flox^–Mx1–Cre–Rosa26^mTmG/+^, RBPJ^flox/+^–Alb–Cre–Rosa26^laz/+^, RBPJ^flox/flox^–Alb–Cre–Rosa26^laz/+^, RBPJ^flox/+^–Mx1–Cre–Rosa26^laz/+^, and RBPJ^flox/flox^–Mx1–Cre–Rosa26^laz/+^ (Fig. 1). All strains were maintained in a C57Bl6 background. Rosa26^mTm G/+^ and Rosa26^laz/+^ reporter mice were used to detect Cre-induced recombination events. The mT gene expresses red fluorescent protein (RFP) and the mG gene expresses green fluorescent protein (GFP) when the Cre sequence specifically recognizes Loxp. Therefore, in the absence of Cre, cells will express RFP and in the presence of Cre expression, cells will express GFP. Therefore, expression of the Rosa26^mTm G/+^ reporter gene can be detected directly with fluorescence microscopy. The Rosa26^laz/+^ reporter gene can be observed with X-gal staining and light microscopy. To induce the Mx1–Cre-mediated deletion of *Rbpj*, 8–10-week-old male mice were injected intraperitoneally with 300 μg/100 μl poly(I:C) (Sigma, St. Louis, MO) four times at two-day intervals. The mice were then injected with the same amount of poly(I:C) four times at one-week intervals (eight injections in total). These reporter genes were driven by hepatocyte-restricted *Alb*- or *Mx1*-promoter-driven transgenes, which express GFP or X-gal in hepatocytes. These mouse lines allowed us to lineage trace the fate of the hepatocytes or progenitor cells.

### Antibodies and primers

The following primary antibodies were used in this study: rabbit anti-mouse Notch4, rabbit anti-mouse Hey1, rabbit anti-mouse RBPJ, rabbit anti-mouse Notch1, rabbit anti-mouse Notch2, rabbit anti- mouse Notch1, rabbit anti- mouse CK7, rabbit anti- mouse CK19, rabbit anti- mouse AFP, mouse anti-goat Hes1, rabbit anti-mouse Notch3 (Sigma), CD133(Abcam).

### Partial hepatectomy (PHx)

Eight-week-old Mx1–Cre RBPJ-KO homozygous mice and heterozygous control mice were fed with 0.04% 2-AFF at 0.6 g/kg per day for five days. One day later, all the mice were subjected to 70% partial hepatectomy (PHx), as described previously^21^. The mice were sacrificed at 1 hr, 3 hr, 6 hr, 12 hr, 1 day, 3 days 4 days, 6 days, 8 days, and 14 days after PHx and the weights of their livers and whole bodies were measured.

### Serum liver enzyme and total bilirubin (TB) assays

Serum levels of alanine aminotransferase (ALT) and total bilirubin (TB) were determined using a standard auto biochemical analyzer (ADVIA 2800, Siemens, Germany).

### Cell culture and generation of stable cell line

HPCs were cultured at 37 °C in Dulbecco’s modified Eagle’s medium (DMEM; Gibco Industries, Tulsa, OK, USA) supplemented with 10% fetal calf serum in humidified air with 5% CO_2_. HPCs that stably expressing RBPJ were generated by transfecting with overexpressing RBPJ plasmids. To inhibit Notch signaling, HPC and HPC–RBPJ cells were treated with the Notch inhibitor N-[N-(3,5-difluorophenacetyl)-l-alanyl]-S-phenylglycine t-butyl ester (DAPT; Merck KGaA, Darmstadt, Germany).

### Liver tissue histopathology

Mouse liver tissues (median and left lobes) were collected, incubated in 4% paraformaldehyde, and embedded in paraffin with traditional methods. Sections (4 μm thick) were cut and stained with hemotoxylin–eosin (H&E) for observation under a light microscope.

### Microfil™ injection

Fourteen days following PHx, mice were sacrifices and we visualized the common bile duct (CHD), tied a suture around it, and injected it with ~1 ml of PBS with a small needle. Microfil™ was subsequently injected into the CHD until the IHBDs were filled with dye. The clearing protocol was performed per the Flow tech website with serial ethanol and methyl salicylate.

### Isolation of HPCs

After the PHx liver tissue were diced, they were treated with dispose (1000 μl/ml; Godo Shusei Co. Ltd., Tokyo, Japan) in 2-[4-(hydroxyethyl)-1-piperazinyl] ethane sulfonic acid (Sigma Chemical Co., St. Louis, MO) and buffered DMEM (Gibco) for 60 min at 37 °C [2]. The liver specimens were then dispersed into single cells by pipetting, and filtered through a nylon mesh filter with a pore size of 132 μm (Nihon Rikagaku Kikai Co., Ltd, Tokyo, Japan). Magnetic M-450 Dynabeads covalently coated with a combination of sheep anti-mouse immunoglobulin G (IgG) and an antibody against rabbit CD133 (Dynal A.S., Oslo, Norway) were used to separate the mouse HPCs, as previously described^6^,^22^. The beads were precoated by incubation in TBS containing 10 mmol/L CaCl_2_ and 1% bovine serum albumin (BSA) to prevent nonspecific binding to the cells. The growth of the liver progenitor cells was measured with a 3-(4,5)-dimethylthiahiazo (-z-y1)-3,5-di-phenytetrazoliumromide (MTT) assay (Promega) and direct counting. HPC apoptosis was measured with flow cytometry.

### Protein isolation and immunoblotting

Total protein was isolated from the mouse progenitor cells or liver tissues from the Mx1–Cre–RBPJ-KO and Alb–RBPJ-KO animals for immunoblotting using 1% sodium dodecyl sulfate (SDS) in RIPA buffer (20 mM Tris-Cl [pH 7.5], 150 mM NaCl, 0.5% NP-40, 1% Triton X-100, 0.25% sodium deoxychlolate, 0.6–2 μg/ml aprotinin, 10 μM leupeptin, 1 μM pepstatin). The protein concentrations of all the lysates were determined using bicinchoninic acid protein assay reagents (BCA method). The nuclear proteins were prepared using the NE-PER Nuclear and Cytoplasmic Protein Isolation Kit (LI-COR Biosciences, Lincoln, Nib), according to the manufacturer’s protocol.

### Cell-cycle analysis by flow cytometry

Cell cycle analysis in 7701 hepatocytes cells HPCs was assessed with flow cytometry. The cells were harvested and immediately fixed overnight in 70% ethanol at 4 °C. The cells were then treated with 50 mg/l RNaseA (Sigma) for 30 min at 37 °C and stained with 50 mg/l propidium iodide (PI; Sigma) for 10 min. The samples were then analyzed for their DNA content using a FACS Aria Cell Cytometer (BD Biosciences, San Jose, CA, USA). The data were analyzed with the Cell Quest software.

### Cell proliferation and viability assay

7701 cells and HPCs transfected with a plasmid encoding RBPJ or mixed with DAPT were seeded on 25 cm^2^ plates to assess their growth. The cells were cultured for 24 h in serum-free DMEM containing insulin (10 mg/ml) for synchronization. MTT (20 μl) was added to each well and culture was continued at 37 °C for 4 h. After removal of the medium, 150 μl of DMSO was added to each well and the optical density (OD) was measured.

### Immunohistochemistry

Paraffin-embedded liver sections (4 μm thick) were immunohistochemically stained. The slides were heated in a microwave at high power in citrate buffer for 10 min for antigen retrieval. The tissue sections were blocked for 20 min, and then incubated with the appropriate primary antibody overnight at 4 °C. The primary antibody was then linked to a biotinylated secondary antibody followed by routine avidin–biotin detection. Diaminobenzidine was used as the chromogen, which produced a brown reaction product.

### Immunofluorescence

Fresh liver tissues collected from the mice were fixed in 4% paraformaldehyde on ice for 1 h. The fixed liver tissues were washed three times with PBS for 5 min on ice before they were dehydrated overnight in 30% sucrose (dissolved in PBS) at 4 °C. The tissues were infiltrated with OCT (Sakura, USA) for 2 h on day 2, and then frozen and stored at –80 °C. Sections (5 μm) were cut with a freezing microtome and stored at –20 °C. With the Rosa26^mTm G/+^ reporter gene, we could observe the tissues directly with fluorescence microscopy. Before analysis, the prepared sections were dried at room temperature for 5 min, after which the OCT was dissolved with PBS for 5 min. The cell membranes were ruptured with 0.2% Triton X-100 at room temperature for 20 min. The nonspecific antigen-binding sites were blocked with 5% BSA and the sections were then incubated overnight primary antibody (1:1000) at 4 °C. After the samples were incubated with anti-rabbit antibody for 30 min on day 2, the cell nuclei were stained with 2-(4-amidinophenyl)-6-indolecarbamidine dihydrochloride (DAPI) diluted 1:1000. All sections were observed with fluorescence microscopy.

### SYBR Green real-time RT–PCR

Total RNA was isolated from the collected liver tissues with TRIzol Reagent (Takara Japan, Shiga, Japan). The RNA was reverse transcribed into cDNA according to the kit manufacturer’s instructions (Takara). Equal quantities of cDNA were continuously amplified by PCR in a 10 μL reaction volume.

### Chromatin immunoprecipitation (Chip)

We selected ATCCCACA and CATGAGAT as the RBPJ binding-site sequences. HPC–RBPJ cells and DAPT-treated cells were fixed by the addition of 1% formaldehyde and used for the following analysis. The cells were incubated at 37 °C, washed twice, scraped, and collected by centrifugation. The cells were then resuspended in SDS lysis buffer and disrupted with sonication. The cell lysates were centrifuged and the supernatants collected and diluted in Chip dilution buffer. The supernatant was then precleared with salmon-sperm-DNA-coated protein A agarose (Sigma), and then immunoprecipitated with 5 μg of anti-RBPJ polyclonal antibody (Santa Cruz Biotechnology) overnight at 4 °C with rotation. The antibody complexes were collected with salmon-sperm-DNA-coated protein A agarose and extensively washed with Chip dilution buffer, according to traditional methods.

### Statistical analysis

All values are expressed as means ± SD. The statistical analysis was performed with two-tailed Student’s *t* test. P values less than 0.05 were considered statistically significant.

## Results

### *RBPJ* KO leads to developmental biliary abnormalities

**Figure 2:**
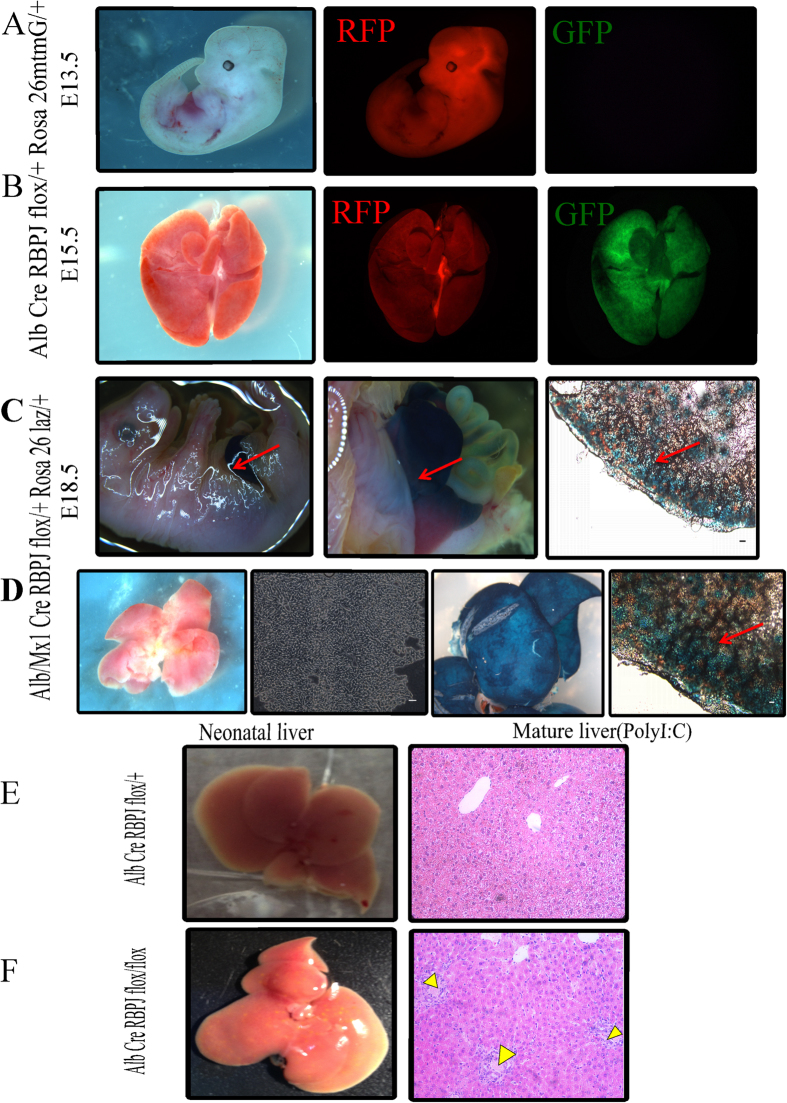
*Rbpj* KO leads to developmental biliary abnormalities. (**A**) In the AlbCre^+^RBPJ^flox/+^Rosa26^mtmG/+^ mouse strain, Alb–Cre is not expressed at E13.5, and thus, there is whole-mount expression of RFP, but GFP is undetectable. (**B**) At E15.5, RFP and GFP were both positive, suggesting that Alb–Cre is becoming active. (**C**) With X-gal staining, AlbCre^+^RBPJ^flox/+^Rosa26^laz/+^ stained positive at E18.5, indicating that RBPJ was successfully knocked out in liver cells. (**D**) Mx1–Cre was induced with poly(I:C) and demonstrates positive staining in liver and hepatocytes. (**E**) Paired littermate studies show that liver-specific *Rbpj*-KO resulted in cholestasis during the postnatal period, bith grossly and histologically. Yellow arrows indicate areas of cholestasis (scale bar: 100 μm).

We constructed multiple congenital liver-specific RBPJ knockout (KO) mice (driven by Alb-Cre or Mx1-Cre) with various reporter genes, specifically LacZ or membrane tomato/membrane green (mt/mG), inserted at the Rosa26 locus. In the LacZ system, cells that are Alb+ or Mx1+ (i.e. liver specified cells) will express Cre that drives both excision of the *Rbpj* gene as well as expression of the reporter, LacZ. In the mt/mG system, cells that are Alb+ or Mx1+ (i.e. liver specified cells) will express Cre that drives both excision of the *Rbpj* gene as well as expression of the green fluorescence reporter EGFP (GFP) while cells without Cre expression will retain the red fluorescent mTomato signal (RFP). To analyze the effect *Rbpj* KO during liver development, we used both Rosa26^laz/+^ and Rosa26^mtmG/+^ as the reporter genes for the direct observation of X-gal staining or for fluorescence microscopy. In the Alb–Cre^+^RBPJ^flox/+^Rosa26^mtmG/+^ mice at E13.5 (prior to albumin induction), the whole mount expresses RFP in all cells and GFP expression cannot be detected in liver (Fig. 2A). In contrast, at E15.5 (during albumin induction), RFP and GFP were both positive in liver, suggesting that Albumin expression was beginning to drive Cre-mediated excision of *Rbpj* (Fig. 2B). Interestingly, we also noted a 50% rate of neonatal mortality in the homozygous KO mice. In the X-gal system, blue liver was seen and individual blue hepatocytes were detected at E18.5, also indicating that RBPJ was successfully knocked out in the liver cells (Fig. 2C). Similar results were seen in the mature liver of Mx1-driven Cre mice following poly (I:C) injection (Fig. 2D). The heterozygous (Alb–Cre^+^RBPJ^flox/+^Rosa26^mtmG/+^) mice showed normal liver development (Fig. 2E). In contrast, the KO (Alb–Cre^+^RBPJ^flox/flox^Rosa26^mtmG/+^) mice showed norrmal morphology in early embryonic development, but during the perinatal period and after birth, there was abnormal development of the bile ducts and emergence of intrahepatic cholestasis (Fig. 2F).

### Effect of liver-specific RBPJ deletion on biliary regeneration

**Figure 3:**
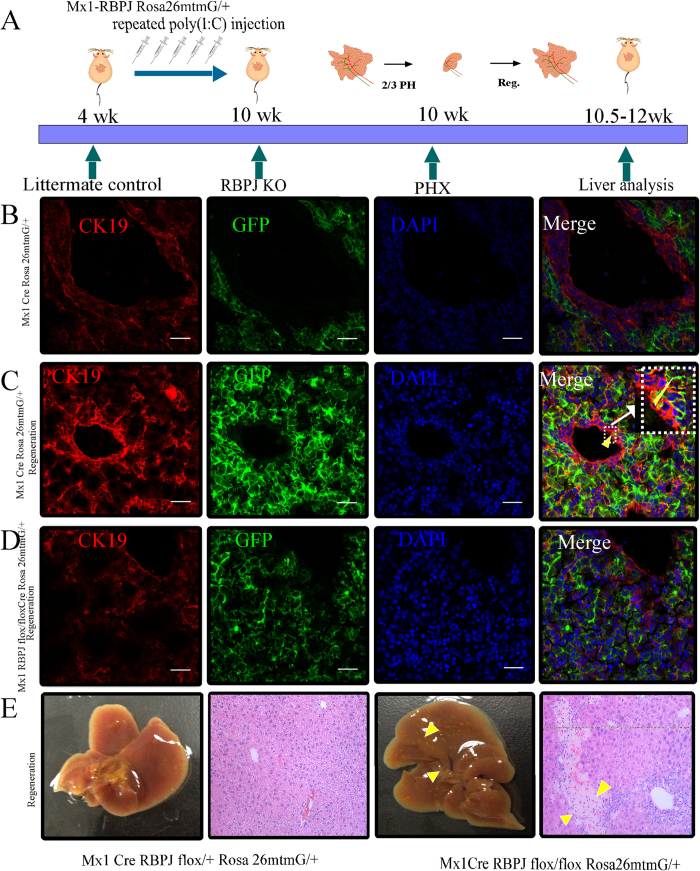
Effect of liver-specific RBPJ deletion on biliary regeneration. (**A**) Mx1–RBPJ-KO mice were induced with poly(I:C) injection. Mouse liver regeneration was established after 2/3 PHx. (**B**) In the normal liver, immunofluorescent staining for CK19 (red) was performed. Rosa26^mTmG^ reporter expression shows GFP-labelled hepatocytes (green). (**C**) In the regenerating liver, immunohistochemical staining for CK19 (red) showed that some regenerating cholangiocytes derived from GFP-labelled cells. (**D**) In the regenerating livers of RBPJ-KO mice, GFP-derived cholangiocytes are absent (scale bares: 200 μm). (**E**) During regeneration, the RBPJ-KO mouse (Mx1–Cre/^+^RBPJ^flox/flox^Rosa26^mtmG/+^) dsiplay biliary abnormalities, intrahepatic cholestasis, and peri-portal necrosis.

**Figure 4:**
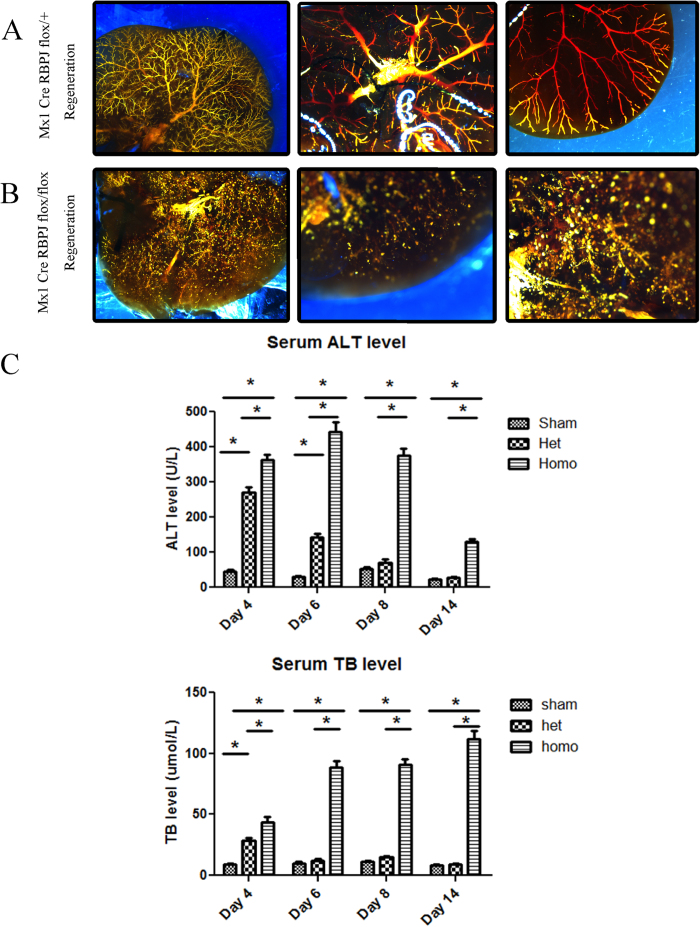
Rbpj KO results in abnormal biliary morphology following regeneration. (**A**) In the regeneration model, the biliary was normal in littermate control mice after Microfil injection. (**B**) In the RBPJ-KO mice, the intrahepatic biliary network filled with Microfil was markedly chaotic and proliferative. (**C**) ALT (U/L) and TB (μmol/L) in mouse sera at different time points.

To investigate the effects of *Rbpj* deletion in the mature liver, we selected the Mx1–Cre recombinase transgene, which can be induced by poly (I:C) injection (Fig. 3A). With poly (I:C) induction, Mx1–Cre was expressed (and RBPJ deleted) in the mature liver. The Rosa26^laz^ or Rosa26^mTmG^ reporter gene was then driven by Mx1–Cre recombinase. Since poly (I:C) was not injected during the developmental period, we found by genotyping analysis that homozygous mice occurred at a probability in accordance with the law of Mendelian genetics and no death was seen in the weaning period. We confirmed *Rbpj* deletion by directly observing Rosa26^mTmG^ reporter expression among liver cells with confocal microscopy (Fig. 3B,C). Expression of the bile duct marker, CK19, was observed with immunofluorescent staining (red). Interestingly, during liver regeneration after PHx, there was regeneration of biliary epithelial cells derived from GFP-labelled cells (Fig. 3C). However this phenomenon was absent in the RBPJ-KO mice during the process of liver regeneration (Fig. 3D). The RBPJ-KO mouse also showed more biliary abnormalities, intrahepatic cholestasis, and peri-portal necrosis compared to littermate controls (Fig. 3E). To evaluate biliary regeneration at earlier time points, we also performed PHx at 1 hr, 3 hr, 6 hr, 12 hr, 1day and 3days and evaluated the expression of the biliary marker, CK7, by immunohistochemical staining. The data shows that RBPJ-KO inhibits the regeneration of CK7 positive biliary structures when compared to littermate controls (Supplementary Figure S1), even at very early timepoints, highlighting the critical importance of Notch signalling in the inititation of biliary regeneration. To further characterize the effect of RBPJ on biliary regeneration, Microfil technology was used. Microfil injection among control mice showed a healthy branching morphology of the intrahepatiic biliary network (Fig. 4A). In contrast, microfil clearly showed a chaotic and disorganized biliary tree in the RBPJ-KO mice (Fig. 4B). Because ALT and TB are biochemical correlates of liver damage, they were used to examine the levels of liver injury and cholestasis during liver regeneration. RBPJ-KO mice showed a significant increase in their serum ALT and TB levels on post-hepatectomy day 6 compared to those of their littermate controls, especially among homozygotes (Fig. 4C). The ALT level was subsequently reduced, whereas the TB level continued to increase to post-natal day 14. This “bilirubin-enzyme separation” is consistent with biliary injury and hepatocyte necrosis with progression toward serious liver failure, and correlates with the histological analysis (Fig. 3E).

### Notch components in liver regeneration

**Figure 5:**
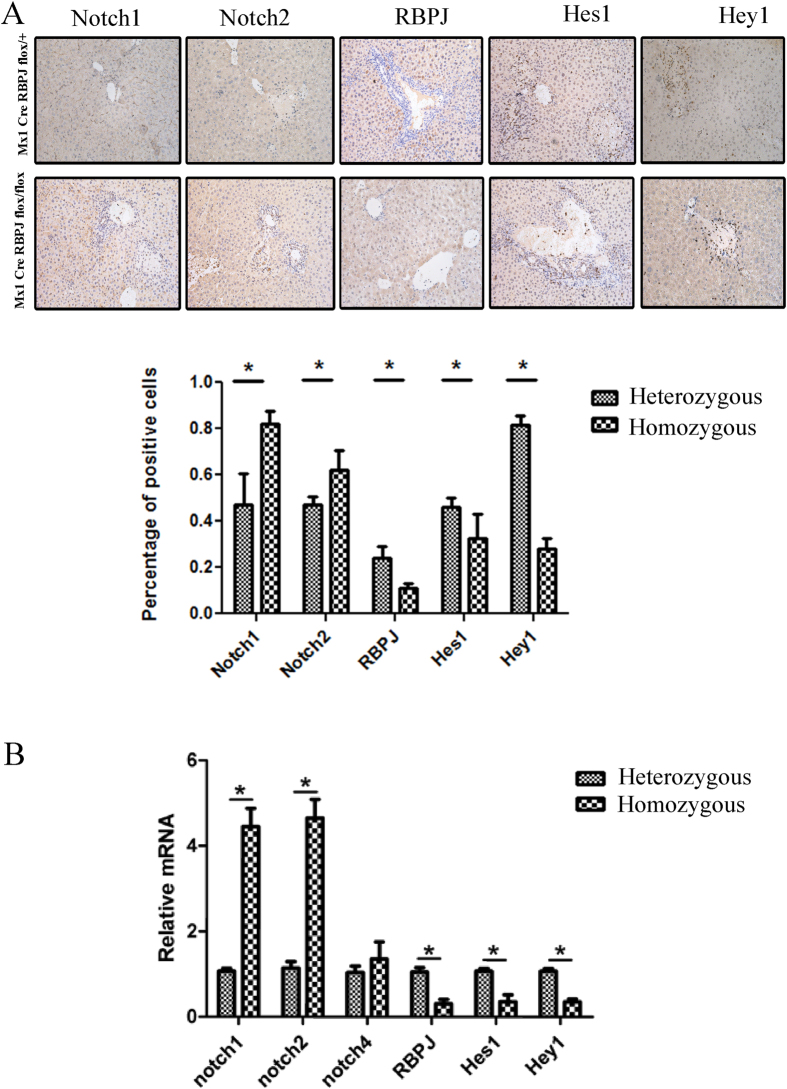
Notch components in liver regeneration. (**A**) Immunohistochemical staining and (**B**) real-time PCR show the expression of Notch signaling components Notch1, Notch2, RBPJ, Hes1, and Hey1in liver tissues. Original magnification: ×200.

To examine the expression of the Notch signaling components during liver regeneration, we measured the stage-specific protein levels for all four Notch receptors, RBPJ, and the downstream targets of Notch signaling, Hes and Hey, using immunohistochemistry (Fig. 5A). We also quantified the mRNA expression levels of these components using real time Q-PCR (Fig. 5B). As expected, RBPJ expression decreased significantly in the RBPJ-KO mice, and was virtually undetectable. The mRNA and protein levels of Notch1, Notch2, and Notch4 were increased in the RBPJ-KO mice compared with that in the littermate control group, presumably as a compensatory mechanism for the loss of RBPJ. The expression of the target genes of the Notch signaling pathway, Hes1 and Hey1, were appropriately reduced in the RBPJ-KO mice, suggesting efficient silencing of the Notch pathway.

### Notch-RBPJ promotes cholangiocyte differentiation from HPCs

**Figure 6:**
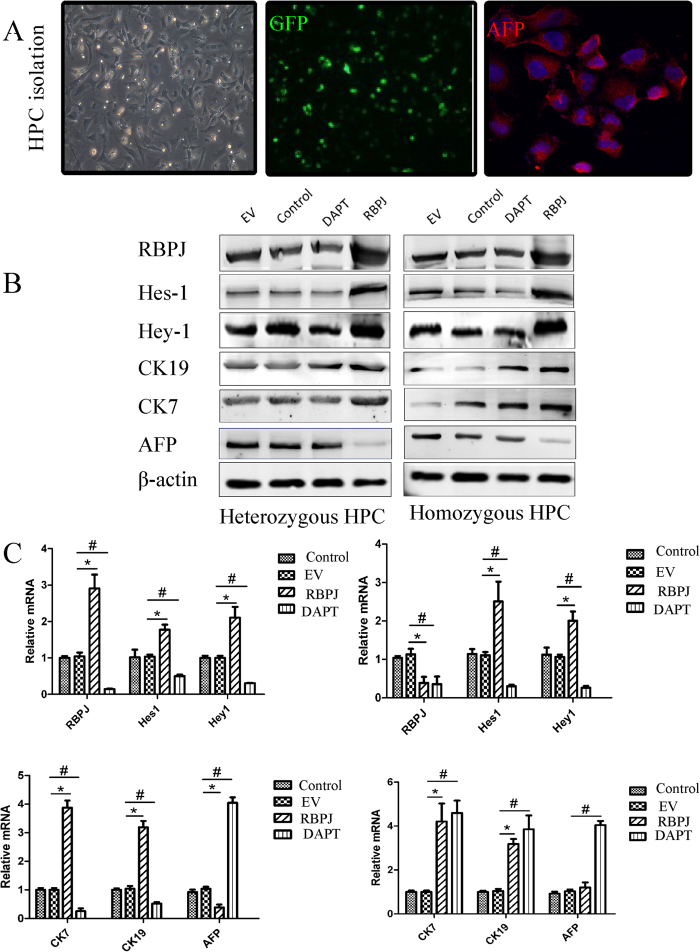
Notch-RBPJ promotes cholangiocyte differentiation from HPCs. (**A**) Isolated HPCs express GFP (Gree) as well as the stem cell marker AFP (red) as detected with immunofluorescence. (**B**) Westerrn blotting on HPCs transfected with RBPJ-overexpression plasmid or treated with DAPT inhition and blotted for components of the Notch signaling pathway: RBPJ, Hes1, and Hey1; cholangiocyte markers, CK7 and CK19, and the hepatocyte marker, AFP. (**C**) Expression of the components of the Notch signaling pathway, cholangiocyte markers, and a hepatocyte marker at the mRNA level as detected by real-time PCR after treatment with RBPJ overexpression or DAPT inhibition.

To clarify the mechanism of Notch–RBPJ in progenitor cell-mediated biliary regeneration, we performed a series of studies *in vitro*. Since previous regeneration studies have suggested that some regenerating cholangiocytes may derive from hepatic progenitor cells, we hypothesized that Notch–RBPJ regulates biliary regeneration by influencing HPC differentiation, proliferation, and apoptosis during regeneration of the adult liver. We isolated HPCs from regenerating mouse livers using immunomagnetic bead isolation^22^,^23^ with an antibody against the progenitor cell marker, CD133. The isolated cells retained their expression of GFP and also stained positive for alpha fetoprotein (AFP), another progenitor cell marker (Fig. 6A). Western blot analysis also confirmed that these cells have prominent EPCAM expression (Supplementary Figure S2). We then transfected these isolated HPCs with the RBPJ-expressing plasmid to detect the dynamic changes in the Notch signaling pathway and the regulatory effects of Notch–RBPJ on differentiation. With an RBPJ overexpression plasmid, the protein (Fig. 6B) and mRNA (Figure C) expression of RBPJ, Hes1, and Hey1 were all increased in isolated HPCs, whereas the differentiation of these components was weakened in the presence of DAPT. When HPCs were transfected with the RBPJ-overexpressing plasmid, their differentiation into bile duct cells, as examined by expression of the cholangiocyte markers CK7 and CK19, increased significantly. Concordantly, expression of the hepatocellular marker, AFP, decreased, suggesting that Notch–RBPJ stimulates the differentiation of HPCs into biliary cells and/or inhibits the differentiation of HPC into hepatocytes, affects that were attenuated after DAPT inhibition of the Notch pathway. Concordantly, in isolated hepatocytes, RBPJ-KO or Notch inhibition enhances the accumulation of the hepatocellular marker, AFP (Supplementary Figure S3).

### Regulatory effects of Notch signaling on the differentiation and proliferation of HPCs *in vitro*

**Figure 7:**
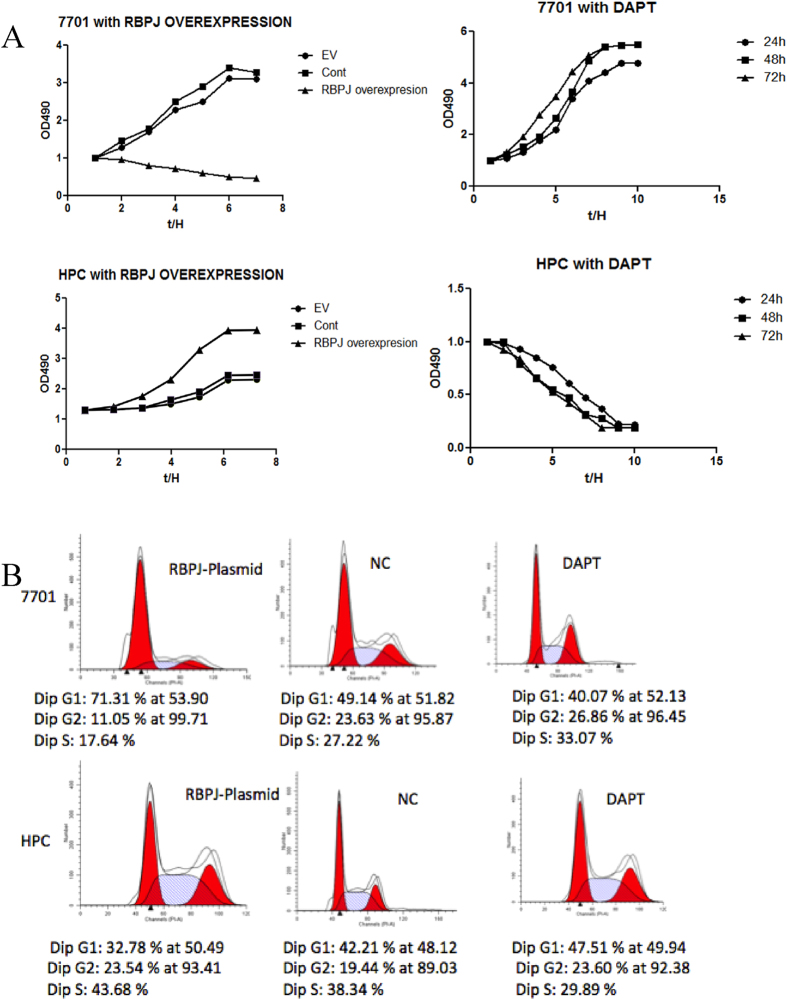
Regulatory effects of Notch signaling on the differentiation and proliferation of HPCs *in vitro*. (**A**) MTT assays to detect 7701 and HPC proliferation with the RBPJ-expressing plasmid or DAPT inhibition. (**B**) Cell-cycle analysis after RBPJ overexpression or DAPT inhibition in 7701 cells and HPCs with flow cytometry. G_1_, first growth phase; S, synthesis phase; and G_2_, second growth phase.

**Figure 8:**
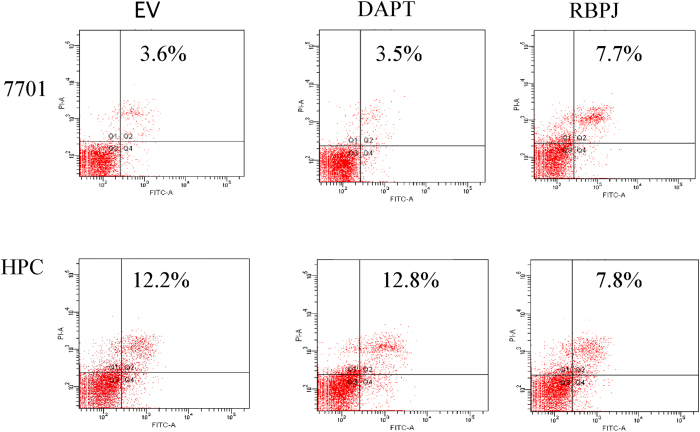
RBPJ overexpression increases hepatocyte apoptosis. Effect of RBPJ on cell apoptosis in different cell types determined with flow cytometry. Q1, necrosis; Q3, normal cells; and Q2 + Q4 means apoptosis. 7701 and HPCs cells were transfected with the RBPJ-overexpressing plasmid or treated with DAPT.

In the 7701 hepatocyte cell line transfected with the RBPJ plasmid, MTT levels decreased, suggesting that RBPJ inhibits the proliferation of hepatocytes. In HPCs transfected with the RBPJ-expressing plasmid, proliferation was increased. The addition of the notch antagonist, DAPT, to 7701 hepatocytes enhanced their proliferation, whereas the proliferation of HPCs was inhibited by DAPT (Fig. 7A). Therefore, RBPJ appears to differentially regulate the proliferation potential of various types of liver cells with a primary proliferative effect on HPCs. We also investigated the effect of RBPJ on the cell cycle with flow cytometry. The overexpression of RBPJ prolonged the G_1_ phase in hepatocytes, which corroborates our interpretation that RBPJ blocks hepatocyte proliferation (Fig. 7B). To evaluate the possibility that RBPJ regulates cell number by inducing apoptosis, we analyzed its effect on apoptosis in 7701 hepatocytes and HPCs with flow cytometry. RBPJ overexpression increased hepatocyte apoptosis, but reduced HPC apoptosis, effects that were reversible with DAPT treatment (Fig. 8). Collectively, these results suggest that RBPJ blocks hepatocyte proliferation by regulating cell cycle progression and promotes HPC proliferation by inhibiting apoptosis.

### RBPJ is recruited to the YAP promoter

**Figure 9:**
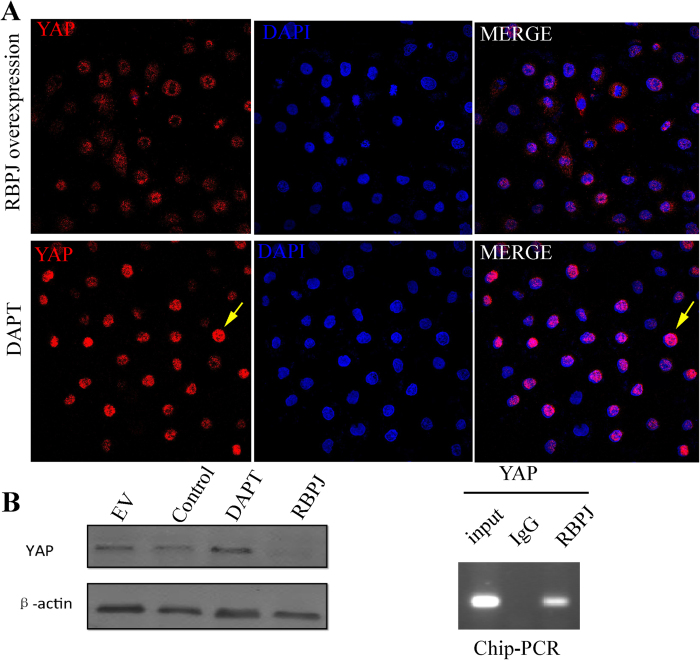
RBPJ is recruited to the YAP promoters. (**A**) HPCs were transfected with RBPJ–overexpressing plasmid or treated with DAPT. Immunofluorescent staining for YAP (red). Western blot for YAP following RBPJ transfection. Chip–PCR was performed with anti-RBPJ antibody for immunoprecipitation and PCR primers targeting the YAP promoter.

We speculated Notch-RBPJ signaling may have inhibitory molecular crosstalk with the Hippo/YAP pathway, which is involved in hepatocyte regeneration^24^ and that the deletion of *Rbpj* would thereby promote the differentiation of HPCs toward hepatocytes. We therefore examined the localization of YAP in HPCs. While both nucleus and cytoplasm stained red for YAP in control HPCs, confocal microscopy showed that YAP was increased in the nuclei of DAPT-treated HPCs (Fig. 9A). To further clarify the relationship between YAP and RBPJ, we performed western blotting in cells overexpressing RBPJ with and without DAPT treatment. This result showed that RBPJ attenuated YAP expression and this effect was reversible with DAPT. DNA immunoprecipitated with an RBPJ antibody was amplified using primers targeting a putative binding site in the *YAP* promoter. A specific positive band was amplified, whereas no positive band appeared in the IgG control, suggesting RBPJ as a repressor of YAP transcription in the modulation of the differentiation potential of HPCs. *In vivo*, we evaluated total YAP and P-YAP by IHC in RBPJ^flox/+^ –Mx1-Cre mice and RBPJ^flox/flox^–Mx1–Cre mice. The results demonstrate increased total and P-YAP levels in RBPJ^flox/flox^–Mx1–Cre mice (Supplementary Figure S4), further corroborating RBPJ as a repressor of YAP.

## Discussion

Liver regeneration includes parenchymal cell regeneration and liver tissue reconstruction. Under normal circumstances, the adult liver is in a highly differentiated and quiescent state. However, with major trauma or cytotoxicity, the liver displays a strong regenerative capacity. The potential mechanisms of liver regeneration mainly include the proliferation of remaining mature liver cells versus HPC activation, proliferation, and differentiation. Early work by Alpini *et al*. demonstrated that, after partial hepatectomy, secretin-induced ductal bile secretion results, at least in part, from proliferation of remaining cholangiocytes^25^. Regardless, the intrahepatic biliary system likely plays an important role in liver regeneration since cholangiocyte proliferation directly regulates the radius of the bile duct and the portal tract is the site of a stem cell niche for HPC. As such, without normal regeneration of the intrahepatic biliary system, liver regeneration will likely be inappropriate. Sparks, *et al*. found that Notch signaling results in disruption of bile duct network^26^. Zhang *et al*. recently found that nhibition of the notch signaling pathway decreases the differentiation of hepatic progenitor cells into cholangiocytes^27^. Our study supports these concepts by demonstrating that interruption of Notch signaling leads to developmental biliary abnormalities as well as abnormal biliary regeneration and altered HPC differentiation through mechanisms involving molecular crosstalk between the Notch and Hippo effectors, RBPJ and YAP.

The Notch signaling pathway is involved in the development of various organs and also plays a central role in the regulation of stem cell differentiation^28^. The disrupted expression of various Notch signaling components is closely related to a variety of human diseases^29^. In the liver, Notch is involved in liver development during the embryonic period, including the development of the bile ducts, homeostasis of the liver sinusoid, epithelial-mesenchymal transitions, and other important biological phenomena^30^. During liver development, Notch signaling is activated in hepatoblasts and controls their differentiation into biliary cells^31^. After liver transplantation, the activation of Notch signaling promotes cholangiocyte proliferation and epithelial-mesenchymal transition^32^. Vanderpool *et al*. found that Notch signaling and HNF-6 regulate mouse intrahepatic bile duct development^33^. Bile duct development requires intact Notch signaling, and in particular, N2 triggers bile duct development. Interruption of Notch signaling causes perforating defects in the intrahepatic bile duct network^34^. Previous studies have also found that Notch2 and Jagged1 mutations are associated with Alagille syndrome, characterized by the abnormal development of the heart, skeletal muscle, liver, and eyes, with defects in hepatic bile duct development and cholestasis in the liver^35^. Fiorotto *et al*. also demonstrated that Notch signaling regulates tubular morphogenesis during biliary repair^36^. However, the role of the Notch signaling pathway in liver regeneration, and especially in bile duct regeneration, is unclear, and the molecular mechanism by which the Notch signaling pathway regulates HPC proliferation and differentiation is also unknown. This study extends the role of Notch signaling to the context of biliary regeneration after hepatectomy where this pathway guides the differentiation HPCs toward cholangiocytes and inhibits YAP-mediated hepatocyte differentiation.

Others have noted in normal wild-type mice that the Notch signaling pathway is highly activated in liver regeneration^37^. Immunohistochemical staining, immunoblotting, and real-time PCR were used to detect the expression of the Notch signaling pathway components, including the related receptors, ligands, and downstream molecules. We found that Notch receptors, such as Notch3 and Notch4, as well as downstream effectors and target genes, including RBPJ and Hes1 were upregulated. These results suggested that the Notch pathway is likely involved in the overall processes of liver regeneration. We designed our subsequent studies to better understand the specific role of the Notch signaling pathway in progenitor cell-mediated biliary regeneration. Because RBPJ is a common downstream transcription factor for all notch receptors, its absence means that Notch signaling is completely blocked. Therefore, we chose *Rbpj* as the primary genetic target. In our experiments, we successfully constructed a multiple liver-specific RBPJ-KO mice using the Cre–LoxP system driven by the liver-specific promotors, Alb and Mx1. We also used this system for lineage tracing studies by incorporating the reporter genes, Rosa26^laz/+^ and Rosa26^mtmG/+^.

We observed abnormalities in liver development after RBPJ-KO, demonstrating in the perinatal period, that the development of the biliary tree was abnormal and cholestasis emerged. We also established that the mortality of the RBPJ-KO mice increased significantly, compared with the heterozygotes, and H&E staining revealed greater necrosis and intrahepatic cholestasis. During liver regeneration after partial hepatectomy, we identified linage-traced cholangiocytes derived from GFP-labelled cells and this occurrence was absent in the RBPJ-KO mice. This suggests some regeneration of biliary epithelial cells derived from either hepatocytes or HPCs that is Notch-dependent. Furthermore, RBPJ-KO mice showed biliary abnormalities, intrahepatic cholestasis, and peri-portal necrosis that was absent in littermate controls. We used Microfil technology to directly observe the derangement of the intrahepatic biliary system in RBPJ-KO mice. After injecting Microfil, we found that the intrahepatic bile ducts in the RBPJ-KO mice were highly disordered, consistent with their biochemical cholestasis. Collectively, these results indicate that disrupting the Notch–RBPJ signaling axis not only disrupts the development of the hepatic bile ducts, but also leads to biliary hypoplasia during normal liver regeneration.

Previous studies have shown that HPCs begin to proliferate and expand rapidly from the portal tract into the hepatic parenchyma when the liver is seriously damaged. HPCs have bidirectional differentiation potential and can differentiate into hepatocytes and cholangiocytes when isolated. Several studies have suggested that during liver development, when hepatoblasts develop into bile duct cells, the Notch signaling pathway is activated and inhibits differentiation into hepatocytes. We wished to determine whether the Notch signaling pathway is also involved in liver regeneration by regulating HPC differentiation. Therefore, we separated and sorted HPCs. These cells were transformed with an RBPJ-overexpressing plasmid or treated with DAPT, an inhibitor of Notch signaling. We then performed various assays to assess HPC differentiation, proliferation, cell cycle progression, and apoptosis. These *in vitro* studies demonstrated that Notch signaling promotes biliary differentiation among HPCs, and inhibits hepatocyte differentiation. RBPJ also appears to also block hepatocyte proliferation by regulating cell cycle progression. Therefore, we concluded that Notch–RBPJ is involved in regulating bile duct regeneration by skewing the HPC lineage toward a biliary fate. This is in line with several studies recently describing stem cell-to-cholangiocyte transitions in the context of induced pluripotent stem cells^38^,^39^,^40^,^41^, all of which require or induce Notch signaling. Concordantly, we observed that blocking Notch–RBPJ signaling during development leads to excessive liver parenchymal regeneration (presumably due to differentiation of hepatoblasts into hepatocytes) along with disordered regeneration of the bile ducts and intrahepatic cholestasis.

To further clarify the molecular regulatory mechanism of Notch–RBPJ in HPC differentiation, and to investigate the possible target genes, we detected molecular crosstalk involving the Hippo and Notch signaling pathways. In particular, western blotting and immunoprecipitation assays demonstrated that RBPJ reduces YAP expression and binds to the promoter region of YAP in HPCs. Furthermore, RBPJ blockade promotes nuclear translocation of YAP. Collectively, these results suggest that RBPJ functions as an antagonist of YAP at multiple levels. Because YAP is known to be an important factor promoting hepatocytes, this serves to inhibit hepatocyte differentiation while promoting cholangiocyte differentiation. These experiments support an overall interaction between the Notch and Hippo signaling pathways at the molecular level that influences the fate decision of HPCs. The specific mechanisms whereby RBPJ and YAP interact will require further study, but may represent a molecular target for interventions aimed at enhancing liver regeneration in various contexts.

**Figure 10:**
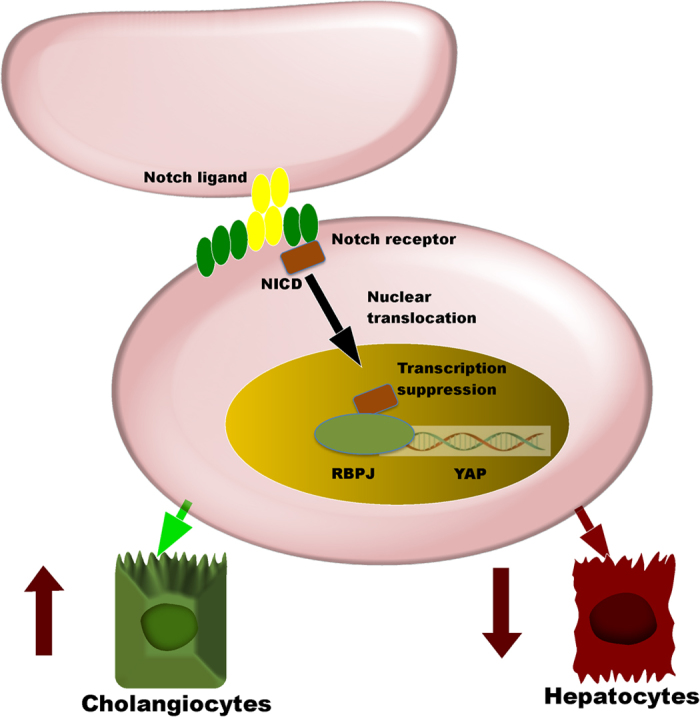
Working model. The Notch–RBPJ–YAP axis as a critical regulator of HPC differentiation to the biliary or hepatocellular fate during liver regeneration.

Increasing numbers of studies have confirmed that the Notch signaling pathway is not only involved in regulating development, as traditionally thought, but also plays an important role in maintaining the homeostasis of adult tissues and cells during the processes of repair and regeneration^29^. In this study, we have clarified the role of Notch–RBPJ signaling in liver regeneration, especially biliary regeneration, and dissected the molecular mechanisms involved. From this study, we have drawn the following conclusions: 1) Liver-specific RBPJ KO leads to abnormal development of the bile ducts during the perinatal period, causing the emergence of cholestasis and increased neonatal mortality; 2) The Notch–RBPJ signaling pathway is involved in liver regeneration, especially bile duct regeneration; 3) Notch–RBPJ regulates differentiation of HPCs by promoting a biliary fate; and 4) Notch–Hippo crosstalk involving RBPJ and YAP may represent a critical regulatory node in HPC differentiation. Overall, these results indicate that the Notch–RBPJ signaling pathway plays a key role in biliary regeneration. This study also extends our understanding of the regulatory mechanisms governing HPC differentiation and provides a platform for new therapeutic targets for the treatment of acute and chronic liver injury (Fig. 10).

## Additional Information

**How to cite this article**: Lu, J. *et al*. Notch Signaling Coordinates Progenitor Cell-Mediated Biliary Regeneration Following Partial Hepatectomy. *Sci. Rep.* **6**, 22754; doi: 10.1038/srep22754 (2016).

## Supplementary Material

Supplementary Information
