# Supplementary material for: Notch Signaling Coordinates Progenitor Cell-Mediated Biliary Regeneration Following Partial Hepatectomy
Source: Sci Rep. 2016 Mar 8;6:22754. doi: 10.1038/srep22754 (PMC4782135; doi:10.1038/srep22754)
Supplement: Supplementary Information [file srep22754-s1.pdf]

Supplementary information

Notch Signaling Coordinates Progenitor Cell-Mediated Biliary Regeneration Following Partial Hepatectomy

Jie Lu [1],Yingqun Zhou[1], Tianyuan Hu [2], Hui Zhang [2], Miao Shen [1] , Ping Cheng [1] ,Weiqi Dai [1], Fan Wang[1],Kan Chen[1],Yan Zhang[1],Chengfeng Wang[1],Jingjing Li[1],Yuanyuan Zheng[1],Jing Yang[1],Rong Zhu[1] ,Jianrong Wang[1], Wenxia Lu[1],Huawei Zhang[1],Junshan Wang[1], Yujing Xia[1], Thiago M. De Assuncao[3], Nidhi Jalan-Sakrikar[3], Robert C. Huebert[3],Bin Zhou [2#], and Chuanyong Guo [1#]

[1]*Department of Gastroenterology, Shanghai 10th People's Hospital, Tongji University School of Medicine, Shanghai, People's Republic of China.*

[2]*Institute for Nutritional Sciences, Shanghai Institutes for Biological Sciences, Chinese Academy of Sciences, Shanghai, People's Republic of China.*

[3]*Division of Gastroenterology and Hepatology; Mayo Clinic and Foundation, Rochester, MN.*

Supplementary Table S1: Primer sequence for genotyping

Name	Forward/Reverse Sequence
Alb–Cre	5′-GCAAACATACGCAAGGGATT-3’,
	5′-AGGCAAATTTTGGTGTACGG-3′
Mx1–Cre	5′-CCGGTCGATGCAACGAGTGATGAGG-3′,
	5′-GCCTCCAGCTTGCATGATCTCCGG-3′
RBPJ$^{flox/+}$	5′-GTTCTTAACCTGTTGGTCGGAACC-3′,
	5′-GCTTGAGGCTTGATGTTCTGTTCTGTATTGC-3′,
	5′-GCAATCCATCTTGTTCAATGGCC-3’
Rosa26$^{laz/+}$	5′-GGAGCGGGAGAAATGGATATG-3′,
	5′-AAAGTCGCTCTGAGTTGTTAT-3′,
	5′-GCGAAGAGTTTGTCCTCAACC-3′
Rosa26$^{mTmG/+}$	5′-CTCTGCTGCCTCCTGGCTTCT-3′,
	5′-CGAGGCGGATCACAAGCAATA-3′,
	5′- TCAATGGGCGGGGGTCGT-3′

Supplementary Table S2: Primer sequence for PCR.

Name	Forward/Reverse Sequence
β-Actin	5′-CATCCGTAAAGACCTCTATGCCAA C-3′,
	5′-ATG GAG CCA CCG ATC CAC A-3′
Notch1	5′-GATGGCCTCAATGGGTACAAG-3′,
	5′-TCGTTGTTGTTGATGTCACAGT-3′
	5′-GATGGCCTCAATGGGTACAAG-3′,
	5′-TCGTTGTTGTTGATGTCACAGT-3′
Notch2	5′-GAGAAAAACCGCTGTCAGAATGG-3′,
	5′-GGTGGAGTATTGGCAGTCCTC-3′
	5′-GAGAAAAACCGCTGTCAGAATGG-3′,
	5′-GAGAAAAACCGCTGTCAGAATGG-3′
Notch3	5′-AGTGCCGATCTGGTACAACTT-3′,
	5′-CACTACGGGGTTCTCACACA-3′
	5′-AGTGCCGATCTGGTACAACTT-3′,

	5′-CACTACGGGGTTCTCACACA-3′
Notch4	5′-GAACGCGACATCAACGAGTG-3′,
	5′-GGAACCCAAGGTGTTATGGCA-3′
	5′-CACTACGGGGTTCTCACACA-3′,
	5′-CACTACGGGGTTCTCACACA-3′
RBPJ	5′-AGTTGCACAGAAGTCTTACGG-3′,
	5′-CCTATTCCAATAAACGCACAGGG-3′
	5′-AGTTGCACAGAAGTCTTACGG-3′,
	5′-AGTTGCACAGAAGTCTTACGG-3′
Hes1	5′-AAA GAC GGC CTC TGA GCA C-3′,
	5′-GGT GCT TCA CAG TCA TTT CCA-3′
	5′-AGTTGCACAGAAGTCTTACGG-3′,
	5′-AGTTGCACAGAAGTCTTACGG-3′
Hey1	5′-CCGACGAGACCGAATCAATAAC-3′,
	5′-TCAGGTGATCCACAGTCATCTG-3′
	5′-AGTTGCACAGAAGTCTTACGG-3′,
	5′-TCAGGTGATCCACAGTCATCTG-3′
CK7	5′-ACGGATGGGGCTAACTTACAA-3′,
	5′-AGTCCTCGATTTGCTCGAACT-3′
CK19	5′-GTTCAGTACGCATTGGGTCAG-3′,
	5′-GAGGACGAGGTCACGAAGC-3′
Alb	5′-CAAGAGTGAGATCGCCCATCG-3′,
	5′-TTACTTCCTGCACTAATTTGGCA-3′
AFP	5′-AGCTTCCACGTTAGATTCCTCC-3′,
	5′-ACAAACTGGGTAAAGGTGATGG-3′
Chip for RBPJ–YAP	5′-CCCACATTTGTTATTTGAGCCC-3′,
	5′-CCTGAACCATCCATCTCATGTTG-3′
	5′-ACATGAGATGGATGGTTCAGGG-3′,
	5′-AGTGAGCACAGGTGAAGGTCAGAG-3′

Supplementary Table S3 Frequency of different genotyping.

	Litter	AlbCre- RBPJ fl/+	Alb Cre- RBPJ fl/fl	Alb Cre+ RBPJ fl/+	AlbCre+ RBPJ fl/fl
Number	30	62	58	64	24
Frequency	/	0.298	0.279	0.308	0.115

Supplementary Table S4 Frequency of different genotyping.

	Litter	Mx1Cre- RBPJ fl/+	Mx1 Cre- RBPJ fl/fl	Mx1 Cre+ RBPJ fl/+	Mx1 Cre+ RBPJ fl/fl
Number	42	70	68	65	66
Frequency	/	0.260	0.253	0.242	0.245

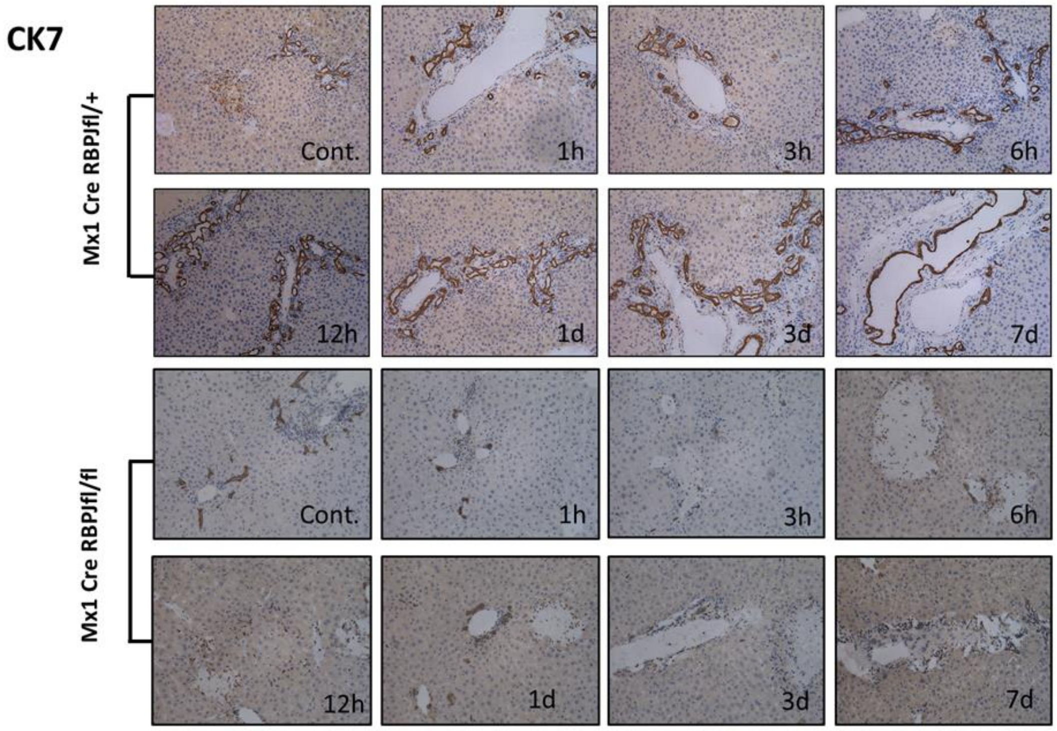

Supplementary Figure S1: CK7 expression in different time points after PHx. In different time points (1hr, 3hr, 6hr, 12hr, 1day , 3days and 7days), IHC staining for CK7 was performed in Mx1 Cre RBPJ $^{flox/+}$ and Mx1Cre RBPJ $^{flox/flox}$.

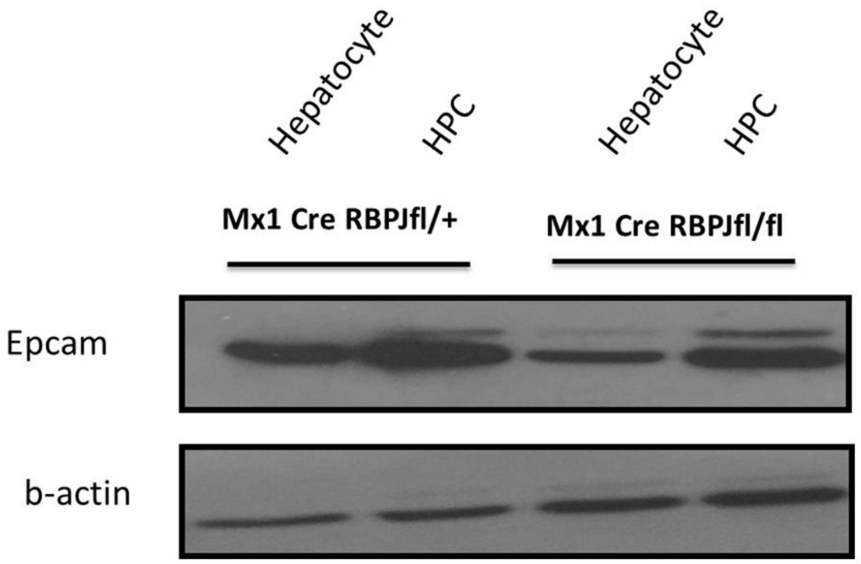

Supplementary Figure S2: Western blotting of EPCAM in HPC and hepatocytes.

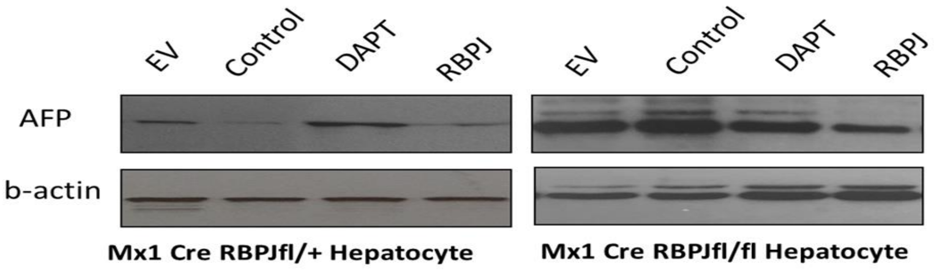

Supplementary Figure S3: Western blotting of AFP in hepatocytes isolated from Mx1 Cre RBPJ $^{flox/+}$ and Mx1Cre RBPJ $^{flox/flox}$ mice.

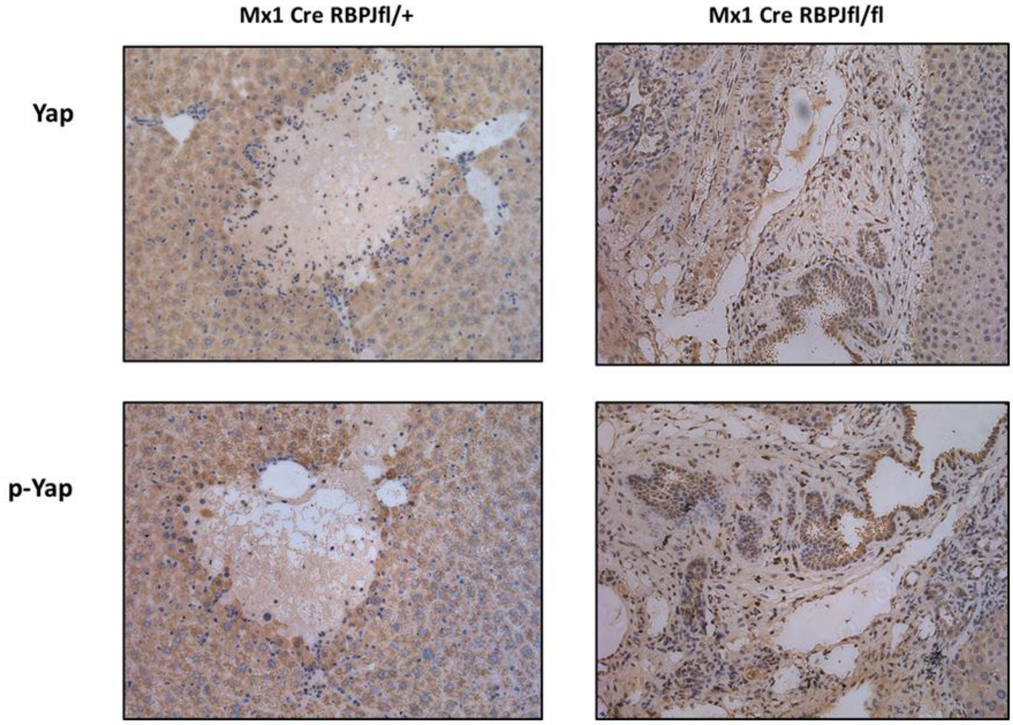

Supplementary Figure S4: IHC staining of YAP and P-YAP in Mx1 Cre RBPJ $^{flox/+}$ and Mx1Cre RBPJ $^{flox/flox}$ mice.
